# Assessment of the Emerging Threat Posed by Perfluoroalkyl and Polyfluoroalkyl Substances to Male Reproduction in Humans

**DOI:** 10.3389/fendo.2021.799043

**Published:** 2022-03-09

**Authors:** Leah Calvert, Mark P. Green, Geoffry N. De Iuliis, Matthew D. Dun, Brett D. Turner, Bradley O. Clarke, Andrew L. Eamens, Shaun D. Roman, Brett Nixon

**Affiliations:** ^1^ Priority Research Centre for Reproductive Science, University of Newcastle, Callaghan, Newcastle, NSW, Australia; ^2^ Hunter Medical Research Institute, New Lambton Heights, Newcastle NSW, Australia; ^3^ School of BioSciences, Faculty of Science, University of Melbourne, VIC, Australia; ^4^ Cancer Signalling Research Group, School of Biomedical Sciences and Pharmacy, College of Health, Medicine and Wellbeing, University of Newcastle, Callaghan, NSW, Australia; ^5^ Centre for Technology in Water and Wastewater, School of Civil and Environmental Engineering, University of Technology Sydney, Ultimo, Sydney, NSW, Australia; ^6^ Priority Research Centre for Geotechnical Science and Engineering, University of Newcastle, Callaghan, NSW, Australia; ^7^ Australian Laboratory for Emerging Contaminants, School of Chemistry, University of Melbourne, Melbourne, VIC, Australia; ^8^ Priority Research Centre for Drug Development, University of Newcastle, Callaghan, NSW, Australia

**Keywords:** male fertility, male infertility, male reproduction, perfluoroalkyl and polyfluoroalkyl substances, PFAS, sperm, toxicants

## Abstract

Per-fluoroalkyl and polyfluoroalkyl substances (PFAS) are a diverse group of synthetic fluorinated chemicals used widely in industry and consumer products. Due to their extensive use and chemical stability, PFAS are ubiquitous environmental contaminants and as such, form an emerging risk factor for male reproductive health. The long half-lives of PFAS is of particular concern as the propensity to accumulate in biological systems prolong the time taken for excretion, taking years in many cases. Accordingly, there is mounting evidence supporting a negative association between PFAS exposure and an array of human health conditions. However, inconsistencies among epidemiological and experimental findings have hindered the ability to definitively link negative reproductive outcomes to specific PFAS exposure. This situation highlights the requirement for further investigation and the identification of reliable biological models that can inform health risks, allowing sensitive assessment of the spectrum of effects of PFAS exposure on humans. Here, we review the literature on the biological effects of PFAS exposure, with a specific focus on male reproduction, owing to its utility as a sentinel marker of general health. Indeed, male infertility has increasingly been shown to serve as an early indicator of a range of co-morbidities such as coronary, inflammatory, and metabolic diseases. It follows that adverse associations have been established between PFAS exposure and the incidence of testicular dysfunction, including pathologies such as testicular cancer and a reduction in semen quality. We also give consideration to the mechanisms that render the male reproductive tract vulnerable to PFAS mediated damage, and discuss novel remediation strategies to mitigate the negative impact of PFAS contamination and/or to ameliorate the PFAS load of exposed individuals.

## Introduction

Perfluoroalkyl and polyfluoroalkyl substances (PFAS) are a diverse group of more than 4,700 synthetic, highly fluorinated, aliphatic chemicals with distinctive chemical properties [see review by Kirk et al. (1)], which render members of this chemical group incredibly stable and environmentally persistent (2, 3). Consequently, PFAS have been employed for a range of purposes including in the formulation of fire-fighting foams as well as in a variety of consumer products (4, 5), such as food packaging, cookware and water repellent clothing (6–9). Since the 1950s, the extensive manufacture, distribution, use and disposal of PFAS has resulted in the widespread environmental contamination and subsequent exposure of humans and animals. Despite endeavors to phase out the toxic eight chain PFAS initiated in 2000, the inherent stability of these compounds has resulted in omnipresence in the global environment (5, 9–11). Thus, many industrialized nations are seeking to implement measures to limit, detect and eradicate PFAS contamination (5, 9). Long-chain PFAS generally have longer environmental half-lives and a high propensity to accumulate in biological systems from which they may take many years to be fully excreted. For example, PFAS such as perfluorooctanoic acid (PFOA) and perfluorooctanesulfonic acid (PFOS) are the most extensively reported long-chain perfluoroalkyl acids described in scientific literature (5) and have a half-life in human serum of 3.8 and 5.4 years, respectively (**Table 1**) (13). Longer chain (≥ 6 carbon atoms) PFAS bioaccumulate to a greater extent than shorter chain analogues (14–17), and also possess longer half-lives (18, 19). Upon entering the body, PFAS bind to albumin in the blood stream and accumulate within the body’s protein-rich tissues (6, 20). Consequently, PFAS are readily detectable throughout the human body as well as accumulating to detectable levels in most bodily fluids, including urine, breast milk, blood, and seminal plasma (21, 22). Notably, in support of the notion that albumin binding is one of the key reasons that PFAS are slowly excreted in urine, Jain and Ducatman have shown that serum PFAS levels decrease under conditions of albuminuria (23). This pathology, during which albumin is able to escape into the urine as a consequence of renal dysfunction, is presumed to result in increased excretion of bound PFAS.

**Table 1:** Summary of a selection of common PFAS chemicals, detailing abbreviations, chemical formula, and half-life in humans.

Chemical Name	Abbreviation	Formula	Half-life in humans
**Perfluorobutane sulfonic acid**	PFBS	C_4_HF_9_O_3_S	28 days
**Perfluorohexane sulphonic acid**	PFHxS	C_6_HF_13_O_3_S	5.3 – 8.5 years
**Perfluorooctane sulfonic acid**	PFOS	C_8_F_17_SO_3_H	3.5 – 5 years
**Perfluorooctane sulfonamide**	PFOSA	C_8_H_2_F_17_NO_2_S	Unknown
**Perfluorobutanoic acid**	PFBA	C_4_HF_7_O_2_	3 days
**Perfluoropentanoic acid**	PFPeA	C_5_HF_9_O_2_	Unknown
**Perfluorohexanoic acid**	PFHxA	C_6_HF_11_O_2_	32 days
**Hexafluoropropylene oxide dimer acid (MS-20244) (Q29388239)**	GenX	C_6_HF_11_O_3_	Unknown (estimated 4 hours to 6 days)
**Perfluoroheptanoic acid**	PFHpA	C_7_HF_13_O_2_	1.2 – 2.5 years
**Perfluorooctanoic acid**	PFOA	C_8_HF_15_O_2_	2.1 – 3.8 years
**Perfluorononanoic acid**	PFNA	C_9_HF_17_O_2_	2.5 – 4.3 years
**Perfluorodecanoic acid**	PFDA	C_10_HF_19_O_2_	Unknown

Table adapted from Fenton et al. (12).

PFOS and PFOA are the two most abundant PFAS found in human serum worldwide (7, 10, 24), with levels of each varying between countries, suggesting differences in the degree of exposure in each country (24, 25). Further, PFOS and PFOA have a propensity of accumulate in our food chains (10) and it is thought that dietary intake is a key pathway of exposure for the general population; either from food packaging or environmental contamination of food products (26–29). Other suggested routes of contamination include household dust (28, 30) or from the consumption of contaminated drinking water (29, 31, 32); although all paths of human exposure remain to be fully identified. Exposure levels vary between locations and individuals and range from background levels in the general population of up to around 14 ng/mL of PFOS and PFOA in the blood (33), through to considerably higher levels in individuals who have been occupationally exposed, or those who reside in contaminated areas (34). The highest concentrations have been detected in individuals employed in PFAS manufacturing facilities with a mean blood concentration of 1,000 to 2,000 ng/mL PFOS and 5,000 ng/mL PFOA (25, 35). Such findings are of particular concern in view of the potential of PFAS to elicit a range of adverse health outcomes.

Here, we review literature pertaining to the emerging threat posed by PFAS exposure, with a specific focus on the male reproductive tract and general male fertility, owing to its utility as a biomarker of general health. Indeed, in what has become a well-established paradigm, male infertility has been shown to serve as an early indicator of a range of co-morbidities such as coronary, inflammatory, and metabolic diseases; conditions that all have associated transgenerational effects (36–42). It follows that adverse associations have been established between PFAS and the incidence of testicular dysfunction, including pathologies such as testicular cancer (43–48) and a reduction in semen quality (35, 49, 50). Accordingly, we give consideration to the mechanisms that render the male reproductive tract vulnerable to PFAS mediated damage, as well as novel remediation strategies to mitigate the negative impact of PFAS contamination and/or ameliorate the concentration of PFAS that has accumulated in exposed individuals.

## PFAS Chemistry

**Figure 1:**
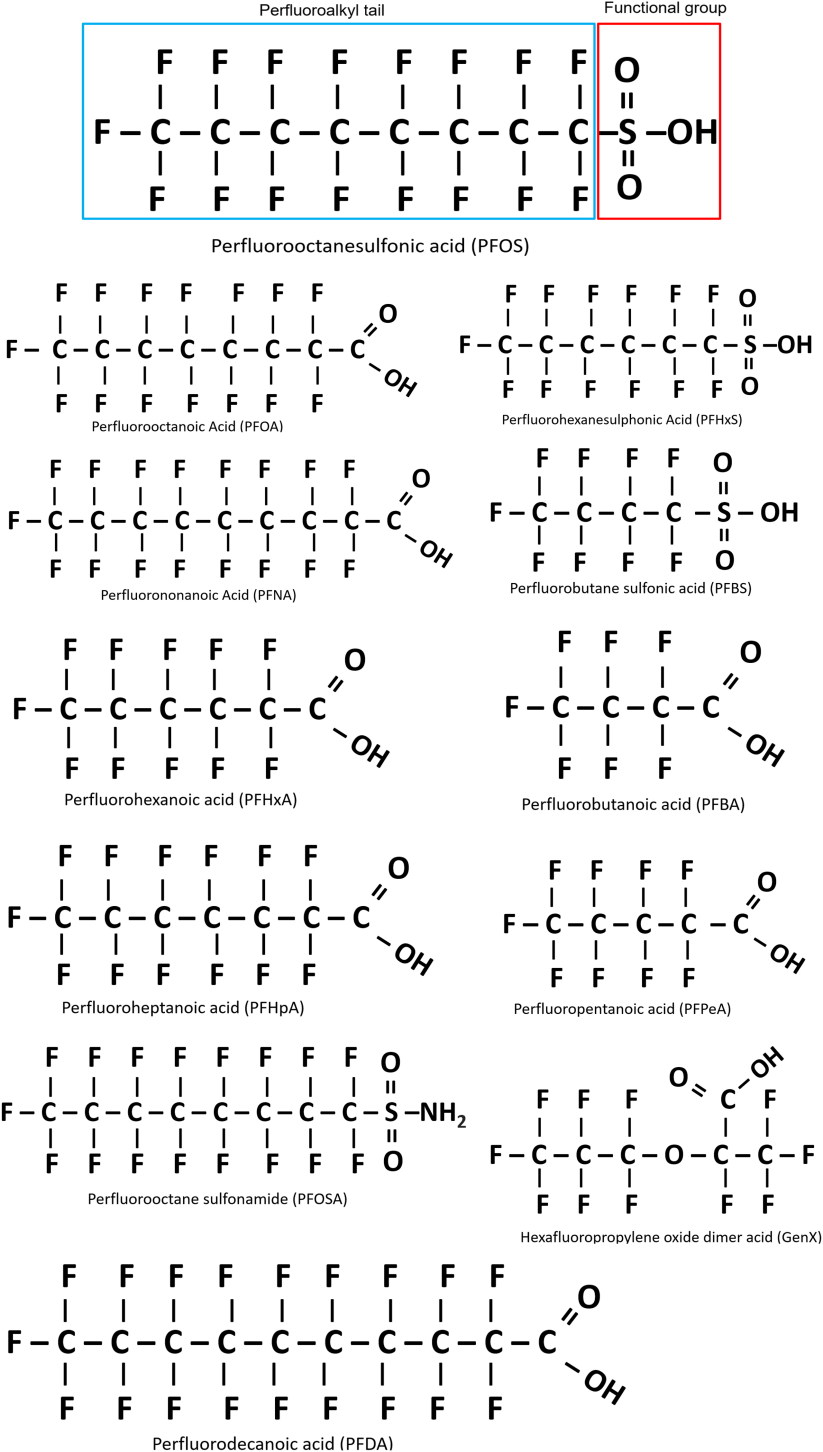
Basic structure of perfluoroalkyl substances (PFAS), using perfluorooctanesulfonic acid (PFOS) as an example. Outlined in blue is the perfluoroalkyl tail (carbon/fluoride chain) and the functional group is outlined in red. All PFAS share these general features, with variation in the carbon chain length and functional group. Figure adapted from Blake and Fenton 2020 (51).

The term ‘fluorinated substances’ encompasses an extensive array of organic and inorganic chemicals that contain a minimum of one fluorine (F) atom, with each substance possessing different chemical, biological and physical properties (3). The properties of each compound are influenced by both the number of F atoms and their position in the molecule, with chemicals classed as partially fluorinated (polyfluoroalkyl), or fully fluorinated (perfluorinated) (4). The most common PFAS are the perfluorinated alkyl acids (PFAAs), which are amphiphilic and exhibit attraction to both aqueous and lipid media, mimicking phospholipid properties. Their structure contains a water-insoluble hydrophobic segment (the fluorinated carbon chain), and a water-soluble hydrophilic functional group such carboxylic acid or sulfonic acid (4). The structural formula of the resulting moiety is C_n_F_2n+1_-R, where R represents the functional group (**Figure 1**) (5). The PFAS moiety contains strong carbon-fluorine bonds conferring unique chemical properties that render these chemicals heat-resistant, water repellent, and exceptionally stable, to the point where they are almost indestructible under normal environmental conditions (5). The fluorination of the hydrocarbon chain drastically changes the chemical properties of the molecule, as the hydrophobic fluorinated segment repels water, while in parallel, the oleophobic properties also repel fat and oil (4). Thus, perfluorinated compounds can effectively lower surface tension and act as efficient surfactants for coatings on non-stick cookware and in food packaging and firefighting foam (1). Individual PFAS are distinguished from each other by 1) the properties of the functional group and 2) the length of the carbon backbone (**Figure 1**). However, PFAS molecules are also further categorized based on their usage, and the history of their manufacture. In this context, group members are described as either legacy PFAS, specifically those molecules with a long history of usage and/or environmental persistence, or as replacement PFAS, which include a new generation of compounds with different chemistries that were designed to replace the original and ‘more’ harmful legacy PFAS (51, 52).

## Routes of PFAS Exposure

**Figure 2:**
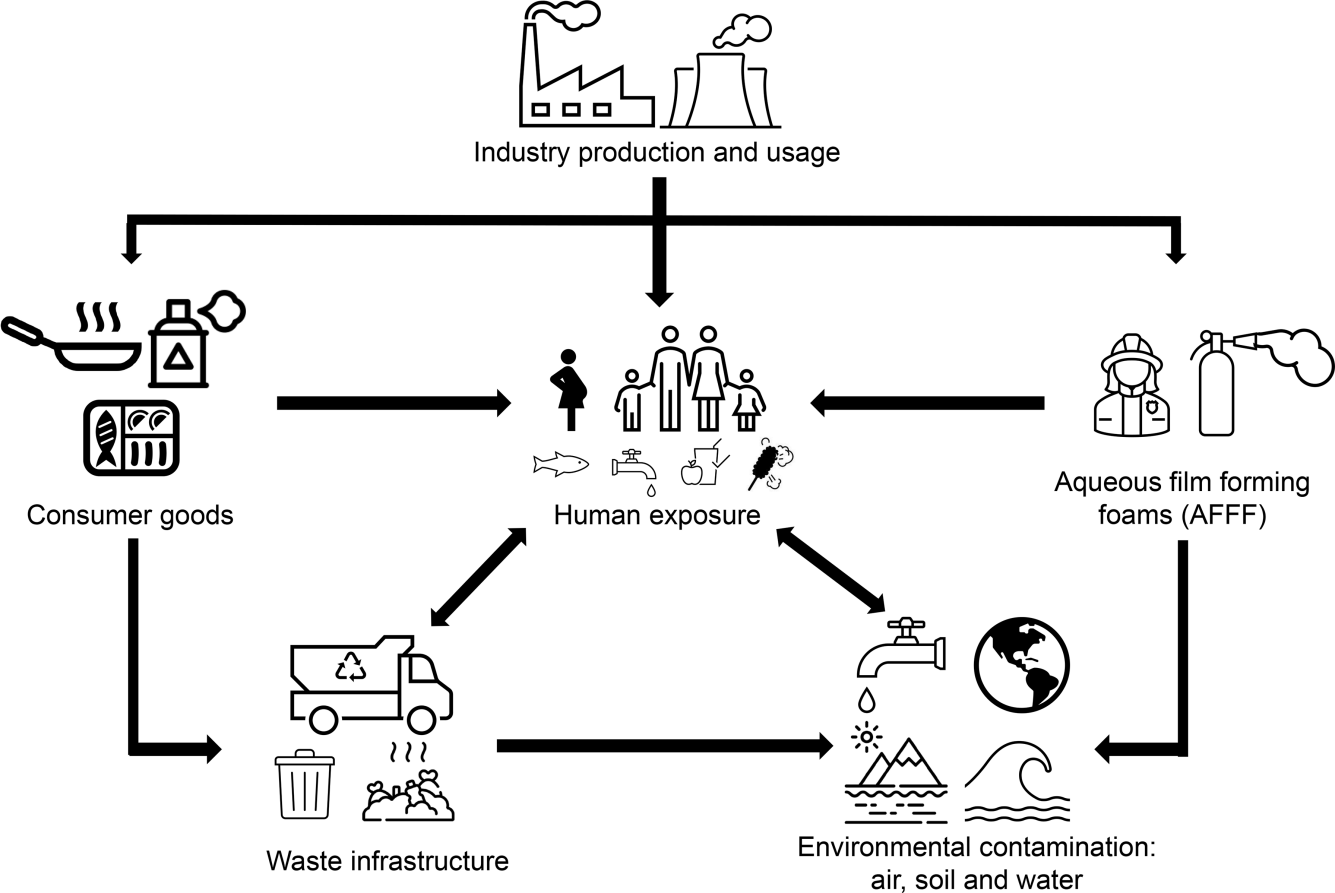
Schematic diagram illustrating the routes of human PFAS exposure. Following production, PFAS are used in consumer products such as food packaging, cookware, water repellent clothing and non-stick fry pans. PFAS are also a main component in firefighting foam, which can leach into the environment, or are otherwise disposed of as industrial waste. Human exposure may occur through use of consumer products or from contaminated water supplies. Accordingly, environmental exposure can occur as a result of waste products contaminating waterways and soil through leaching of firefighting foam and waste from industry and consumers.

PFAS exposure can arise through several routes (**Figure 2**), with environmental contamination occurring at varying stages of production, usage, and waste disposal. In particular, PFAS have found application as a major component of aqueous film forming foams (AFFF) (53), which are widely used for firefighting, military training activities and at airports and thus has resulted in extensive contamination of nearby soil and waterways (31, 34, 54, 55). Elevated PFOS/PFOA levels have been detected in the serum of individuals living in areas with high levels of these chemicals in their drinking water (11, 31), including those communities located in close proximity to military bases, airports and PFAS manufacturing factories (31, 56). Not surprisingly, greater plasma contamination levels have also been detected in occupationally exposed individuals such as firefighters and factory workers manufacturing or using PFAS (31, 34, 55, 57, 58). Industry waste and AFFF usage has resulted in widespread contamination of groundwater, often used as drinking water, with dietary exposure suggested to be the main route of exposure for adults (29, 34, 56, 59). In addition, background levels of contamination are seen in the general population who are exposed to PFAS through drinking water (29, 60), house dust (61) and food consumption (27, 62, 63), with the latter arising from the extensive use of PFAS in consumer packaging. Compounding this situation, prenatal exposure can occur through the placenta (64, 65) and young babies can be exposed through breast milk (66, 67).

## Accumulation and Distribution of PFAS in the Body

PFAS enter the body through ingestion (31), inhalation (68) or dermal exposure (69). Once they have entered the bloodstream through gas-exchange or digestion, PFAS bind to serum proteins such as the major transport protein, human serum albumin (HSA) (70, 71). It appears PFOS has a greater binding affinity for HSA than PFOA, which correlates with the known longer half-life of PFOS (**Table 1**) (12, 72). Due to their biochemical stability, PFAS chemicals tend to accumulate within the body (1) and move from plasma into tissues, with the highest levels being found in human tissues with a larger blood supply such as the liver, lungs and kidneys (6, 20, 66). This is also reported in studies of mice where the highest accumulation of PFOS has been documented in the liver, lungs, kidney and bone marrow (73), as well as in primates, with the kidneys and blood also showing higher levels in comparison to other tissues (66). Notably, the tissue distribution of PFAS is influenced by multiple factors including species and gender, chemical characteristics such as chain length and functional group, as well as exposure dose (66, 74, 75). Most environmentally relevant PFAS have chain lengths between 4 and 13 fluorinated carbons (15, 76) giving rise to a variety of different structures such as branched forms (4, 76), although the most commonly detected PFAS in humans and wildlife are linear forms (**Figure 1**) (77). Longer chain PFAS have a greater potential to accumulate in living organisms than do shorter chain PFAS (<6 carbons) (14) due to the ability of longer chain PFAS such as PFOA, PFOS and perfluorohexanesulphonic acid (PFHxS) to bind to a wider range of serum proteins, including transferrin, plasma gamma-globulin and albumin (70). This is supported by evidence that PFAS accumulation occurs in protein-rich tissues such as the liver (66). Such evidence has led to the replacement of legacy and long-chain PFAS with structurally similar shorter chain variants, thought to be less toxic; for example, perfluorobutane sulfonic acid (PFBS) has been used to replace PFOS (51) and GenX (hexafluoropropylene oxide dimer acid) has replaced PFOA (16). Plasma concentrations support this rationale with longer chain PFAS such as PFOA and PFOS showing higher levels [3.9 and 20.7 µg/L, respectfully (21)] in comparison to the shorter chain replacements PFBS [typically below the detection limit of 4.2 µg/L (9)] and perfluorobutanoic acid (PFBA) [3.3 µg/L (78)]. However, little is currently known about the toxicology of these replacement chemicals (79). Additionally, bioaccumulation of PFAS appears to be influenced by the functional group(s) attached to the hydrocarbon backbone of each PFAS molecule. For example, compounds that harbor an attached carboxylic acid functional group have been shown to accumulate less in fish, than those with a sulfonate functional group of the same carbon chain length (14, 18). Although the exact mechanisms behind this disparity are currently unknown, it may be due to the stronger affinity for proteins seen with longer chain length and in sulfonic acids (71).

## PFAS Human Health Associations

Increasing awareness of the dangers of PFAS and their propensity to bioaccumulate has led to a surge in scientific research and public interest, with PFAS being labelled as a potential risk for humans and the environment by the Scientific Committee on Health in 2018 (80). Studies have been conducted in both human and animal models to investigate possible health consequences arising from PFAS exposure (1, 43). The most commonly investigated PFAS with regards to human health are PFOS and PFOA (81), with a range of additional PFAS having been studied including PFHxS and PFBS (1) (**Table 1**). Mounting evidence from these studies supports an association between PFAS and an array of human diseases and disorders (1). However, it is somewhat difficult to definitively link causality to PFAS due to the variation in chemistries and potential biological activities between the different classes of PFAS, the duration and degree of exposure, potential synergistic or antagonistic effects of PFAS combinations in the body as well as the often-overlooked precursors of PFAS, which degrade to the terminal perfluoroalkyl acids (PFAAS) (82, 83). This situation is further compounded by the mechanisms of PFAS exposure, which vary both between and within communities, resulting in distinct PFAS profiles among individual subjects. Furthermore, disparities also exist in an individual's genetic and phenotypic constitution within affected populations, which could ultimately influence their PFAS clearance rates and susceptibility to the biological effects of these chemicals. Notwithstanding these limitations, the balance of evidence supports the potential for PFAS exposure to elicit adverse health sequelae at differing developmental stages and ages (66, 84–88). The C8 Health Project also bears out this conclusion; a comprehensive investigation of an entire community of 69,000 people exposed to PFAS *via* consumption of contaminated drinking water (43). This study revealed probable links between PFOA exposure and six diseases: kidney and testicular cancer, thyroid disease, high cholesterol, ulcerative colitis, and pregnancy-induced hypertension (43), findings that are supported by the International Agency for Research on Cancer evidence (48, 85). Although, it should be noted this evidence relates to PFOA exposure only, and therefore, further investigations of this nature are required for the remaining range of PFAS.

Building on this evidence, the greatest and most consistently reported metabolic consequence of PFAS exposure is dyslipidemia, with several notable studies finding links between serum PFAS and dysregulated lipid profiles (89), including increased low-density lipoprotein (90, 91), triglycerides (92) and total cholesterol (90, 91, 93, 94) in addition to diminished high-density lipoprotein (89). However, the extent of cholesterol dysregulation is variable across PFAS exposure levels as is the response to different forms of PFAS; with PFOA and PFOS demonstrating the most consistent effects between studies (62).

Epidemiological evidence has also linked PFAS exposure to the prevalence of testicular cancer, with the International Agency for Research on Cancer concluding PFOA is possibly carcinogenic to humans (48) and the United States Environment Protection Agency declaring it a likely carcinogen (95). In this context, studies by Barry et al. reported a strong association between testicular cancer and PFOA exposure (hazard ratio of 1.34) in adults exposed through drinking water assessed as part of the C8 Health Project cohort (43, 44). Similarly, Vieira et al. identified a positive correlation in individuals exposed to very high PFOS levels, with an adjusted odds ratio of 2.8; although a potential limitation of this study was the relatively small sample number (45). Confounding this situation, three additional studies focusing on PFOA exposure and mortality from testicular cancer all failed to identify an association in occupationally exposed workers (87, 96, 97). Thus, whilst not universally demonstrated, the potential significance of these positive associations is highlighted by parallel correlations between the widespread increase in worldwide PFAS usage and the rising prevalence of testicular cancer; a pathology that has significantly increased in recent times to become the most common malignancy in young men aged 20-40 years (98–101). Although the characterization of testicular cancer remains incomplete, there is speculation that environmental factors, as opposed to genetic factors, are a key contributor to the etiology of this form of cancer (98, 102).

## Difficulties Associated With the Study of the Effects of PFAS Chemicals on Human Health

Many challenges exist that have hindered attempts to fully assess PFAS effects on health, including those directly related to tracing the mode and levels of PFAS exposure in the general population, consequences of PFAS precursors, compound effects of PFAS mixtures, as well as nuances specific to studies of animal models (103). The use of the latter has proven invaluable for studying the toxicology of PFAS exposure, albeit with variable outcomes (66, 104, 105). Laboratory rodents are the chief animal model employed, with zebrafish also being utilized in recent times, particularly in the context of assessing the impacts of PFOA and PFOS exposure (12). Amongst animal models, considerable interspecies variation has been noted, but thus far, the mechanistic basis of such inconsistency has not been entirely resolved. Known variables include biological mechanisms of PFAS action within target tissues, rates of PFAS metabolism and elimination, as well as assessment of different disease endpoints (12, 29, 51, 103). For instance, the half-life of PFOA in mice, at 6 days, is much shorter than in rats at 16 to 22 days and is generally significantly longer in humans (~2.1 to 3.8 years) (12). These differences are further confounded by differential responses between genders, with PFOA being eliminated much quicker from female rats (2-4 hours) compared to their male counterparts (4-6 days) (12, 106, 107). Li et al. (108) have reported similar results in humans with a significantly lower PFOS half-life being seen in women (3.1 years) compared to males (4.6 years). Similarly, Zhang et al. (109) reported that PFOS half-life was shorter in women under 50 years of age (6.2 years) in comparison to women over 50 and for males of all assessed age groups (27 years), although, the same trend was not seen with PFOA. However, it should be noted that this estimated half-life of 27 years for PFOS is higher than that calculated in similar studies, and as such, should be considered an upper limit estimation.

The manufacture and pervasive use of PFAS began in the 1950s (4), meaning that virtually all humans born after this time have potentially been exposed to some degree of PFAS contamination. As a consequence, there is a genuine difficulty in identifying a naïve unexposed control cohort, a situation that hinders the reliability of epidemiological models and cohort studies used to compare exposure groups and evaluate the risk of disease associated with varying PFAS exposure levels (51). Further, direct evaluation of the health outcomes of an individual resulting from PFAS exposure provides challenges due to the wide array of PFAS profiles detected in individuals within the same community, and between geographically distinct communities (51). Indeed, an individual’s PFAS profile depends on several variables, such as the source of exposure, which can range from contamination associated with standard food packaging, through to the waste products encountered within the vicinity of a facility which manufactures PFAS (51). Exposure sources are also likely to differ over time due to fluctuations in PFAS usage as has been seen with the progressive phasing out of PFOS and PFOA chemicals in favor of alternative short chain PFAS derivatives (51). Additionally, possible synergistic or antagonist effects between different PFAS molecules may result in variable health outcomes between individuals (51), yet little is currently known of the repercussions of such chemical interplay (66). Variations in exposure also occur throughout the lifetime of an individual and can range from consistent chronic exposure to intermittent shorter periods of exposure. This exposure range has apparent consequences for an individual’s PFAS profile making attempts to relate PFAS exposure to incident health outcomes difficult. Indeed, PFAS-related health outcomes emerging in adults may be attributed to exposure at one or more key stages of development such as the *in utero*, childhood or puberty stages (110), or alternatively, to chronic life-long exposure. Additionally, it cannot be determined whether an individual’s PFAS contamination level is a result of a current exposure(s) or accumulation over a period of several years.

Another limitation of PFAS investigation is knowledge of the full assortment of contaminating PFAS chemicals. Initial identification of these chemicals in human serum was reported in 1980 when PFOA was discovered in a group of industrial plant workers exposed to fluorochemicals (111). A subsequent reduction in the manufacture, and phasing-out the use of PFAS classed as being damaging agents began in several countries in 2000 (5). Consequently, the original PFAS were replaced with other chemical analogues thought to be less toxic and which did not accumulate as readily in biological systems (79). Regrettably, the next generation of PFAS has subsequently been found to be detrimental to human health, with a prominent example being GenX, a branched short-chain PFAS that has subsequently been found to be more toxic than the PFOA it replaced (16). As a result, new PFAS are constantly being added to the list of hazardous and toxic chemicals (112, 113), which emphasizes the necessity for increased investment into PFAS-related research.

Currently, determination of the PFAS profile of an individual is limited by available testing methods. Serum testing is performed most often using mass spectrometry paired with liquid chromatography (78). Mass spectrometry identifies organic compounds based on their molecular mass and can detect and quantify compounds with a high level of sensitivity. Many methods measure a limited subset of 20-30 PFAS, which potentially leads to inaccurate PFAS profiles, and should be considered when comparing PFAS studies (114). The limit of detection varies between PFAS (e.g., 1.10 ng/L for perfluoroheptanoic acid (PFHpA) to 25.1 ng/L for PFBA), with the limit of quantitation being even greater (e.g. 3.3 ng/mL for PFHpA, to 75.3 ng/L for PFBA) (78). If a particular PFAS is present below the method reporting limit in a sample it would be assumed not to be present in the assessed sample, thus introducing inaccuracies in the assignment of biological effects. These limitations in measurement techniques highlight the need to identify and understand new and emerging fluorinated compounds to allow an accurate determination of exposed communities and the efficacy of remediation strategies to reduce exposure.

The confounders documented above highlight the requirement for reliable markers of general health with which to determine the risk posed by PFAS exposure. Here, we explore the utility of employing male reproductive health as one such indicator to understand the molecular pathways by which PFAS drive pathophysiological responses, a strategy that builds on evidence that the male germline is vulnerable to a variety of environmental toxicants (1, 115).

## The Relationship Between Male Infertility and Overall Health

Infertility is a reproductive system disease that impacts 16 to 25% of couples, with almost half of all cases attributed to male reproductive issues (116). Such problems are often connected to semen abnormalities, key contributors to which include body mass, lifestyle, age, and environmental exposures (116). Over the past few decades, decreasing trends in semen quality have been reported but there remains no clear explanation for the underlying causes of this decline (116). In recent years there has also been increased understanding that the general health of a male is closely related to his reproductive health (116), with strong associations established between male infertility and future health; especially the development of testicular cancer (117–120), and chronic non-malignant diseases such as ischemic heart disease and diabetes (36, 37, 40, 42, 116, 121). It has been proposed that shared genetic pathways, lifestyle factors and the environment, possibly acting *in utero*, could play a key role (122). Mounting evidence implies that semen quality can serve as a biological marker for future male health, as multiple epidemiological studies of notable size (>50,000 men) describe consistent associations between reduced semen quality and mortality. For instance, diminished semen parameters, such as sperm count, concentration, motility, and morphology, are related to a 2.3-fold greater risk of death in the following eight years: factors comparable to the risk of death due to diabetes or smoking (36, 42, 117). These studies reveal that men with atypical semen characteristics commonly die due to a higher prevalence of testicular cancer and/or altered androgen signaling, which culminate in the onset of metabolic, cardiovascular, or inflammatory diseases (123, 124); a disease set not too dissimilar to those suggested to onset due to PFAS exposure (1, 43).

Hence, current epidemiological evidence aligns with the association between male infertility and PFAS exposure, as seen with the link between male infertility and risk of chronic disease and mortality. Nevertheless, the scarcity of prospective studies and insufficient adjustment of confounders hinder the ability to ascertain the causality of these associations, and the pathogenic pathways linking these conditions are still ambiguous (1). Despite this, male fertility, particularly the clinical assessment of basic sperm parameters, allows for readily accessible biomarkers and presents as a potentially important resource to identify diseases promptly and predict the long-term health of an individual (36, 39–42). The theory that male reproductive pathologies are triggered by environmental exposure is not a new concept. Indeed, several studies have investigated a range of environmental contaminants (115) such as pesticides and herbicides (125), acrylamide (126), and radiation (127) and their implications for male fertility. In addition, these studies provide an important precedent for equivalent research to be performed on PFAS.

## Known Effects of PFAS Exposure on Male Fertility

Despite the publication of several studies exploring the relationship between PFAS exposure and male fertility, the evidence presented is often conflicting (81, 128), and further such studies are hindered by the nature of the chemicals assessed and the pre-existing history of worldwide PFAS exposure. Notwithstanding the limitations imposed by these confounders, male ailments such as testicular cancer are perceived as a prominent endpoint of PFAS exposure (1, 43–47) (**Table 2**). Further evidence of testicular dysfunction is supported by large cohort studies assessing semen quality (49, 140). In this context, a dose-response relationship may exist between chronic PFOA and PFOS exposure and sperm production. A 35% decline in normal sperm production was observed in the upper tertile of PFOS concentration (> 27.3 µg/L), compared to that of the first tertile (< 11.9 µg/L) (35). Similarly, a 40% decrease in normal sperm production was recorded in high PFOS and PFOA exposed individuals, compared to men classed as having low exposure levels (129). Moreover, *in utero* exposure to PFOA was shown to lower total sperm count (130). Further to this, recent studies have described a significant association between PFAS exposure and several indicators of human sperm quality (49, 134). For instance, Toft and colleagues (35) found no consistent associations between exposure to multiple PFAS and sperm concentration and count, or semen volume but did record a decrease in sperm cells with normal morphology in association with higher PFOS levels (35). Similarly, Joensen et al. reported equivalent results in their 2009 study with PFOA and PFOS (129), but these outcomes could not be reproduced in their later study published in 2013 (131), possibly due to lower levels of PFAS recorded in this later cohort (0.5% for PFOS), in which no participants were classified in the ‘high exposure’ category as per the criteria in the first study. Further studies also failed to find an association between PFAS and sperm quality (132, 133, 135–137); thus precluding the establishment of causative links (133, 136). Sperm motility has also been found to correlate both positively and negatively with PFOA exposure. One study investigating the implications of PFAS exposure on sperm parameters found greater sperm motility the study cohort with the highest serum PFOA levels (35). However, this observation was not consistent across all countries, nor the individual PFAS examined, and due to the many statistical tests performed in this study, the authors concluded such results might be due to chance events alone (35). In contrast, an independent study reported a significant negative correlation between several PFAS in semen, including PFOA, and sperm motility (78). This latter study also found associations between the PFAS concentration in semen and sperm motility, which indicates that seminal concentrations of PFAS may be more indicative of semen quality than that of serum PFAS levels. Such a finding has implications for the accuracy of extrapolating data from different sample sources across studies. Thus far, less than 15 studies have been conducted to investigate associations between PFAS levels and sperm parameters (1, 141) (**Table 2**). Half of these studies show no association while the other half report some associations. Yet, no studies consistently found the same set of altered sperm parameters due to PFAS exposure. Differing results may, in part, be attributed to the variation in the studied cohorts between countries; for example European and Arctic (35), versus Chinese populations (78), wherein participants are likely to have been exposed to different PFAS profiles depending on the source and route of contamination, not to mention other environmental factors to which they may be exposed, that could act synergistically or antagonistically. Although the variation in outcomes reported in these studies highlights the difficulties in directly comparing PFAS studies, the existence of positive correlations between PFAS exposure and abnormal sperm characteristics uphold the view that internalized PFAS do localize to the testis, along with other organs of the body, thereby forming a useful model to study PFAS-induced damage. However, it should be noted that using sperm parameters as a measure is quite a blunt tool and small changes are unlikely to be informative due to the wide variation seen between males and the low threshold for WHO defined parameters (142). Therefore, researchers should exercise caution when interpreting data on sperm parameter changes.

**Table 2:** Summary of outcomes from studies investigating the impact of PFAS on human male reproductive function.

Assessed outcome	Serum PFAS assessed	Timing of PFAS exposure	Outcome	References
**Prevalence of testicular cancer**				
	PFOA PFHxS	Adulthood	Increased	Frisbee et al. (43) Barry et al. (44) Kirk et al. (1) Vieira et al. (45) Bartell and Vieira (47)
	PFHxS	*In utero*	Increased	Lin et al. (46)
**Sperm morphology**				
	PFOS, PFHxS PFOA + PFOS PFOSA	Adulthood	Decrease in percentage of normal spermatozoa	Toft et al. (35) Joensen et al. (129) Louis et al. (49)
	PFOA, PFOS	*In utero*	No change	Vested et al. (130)
	Multiple PFOS, PFOA, PFNA, PFHxS	Adulthood	No change	Joensen et al. (131) Petersen et al. (132)
**Sperm count and concentration**				
	PFOA	*In utero*	Decrease in sperm count and concentration	Vested et al. (130)
	PFOS, PFOA, PFNA, PFHxS	Adulthood	No change	Toft et al. (35) Joensen et al. (131) Petersen et al. (132) Raymer et al. (133)
	PFOS	*In utero*	No change	Vested et al. (130)
**Sperm DNA quality**				
	Multiple	Adulthood	Increased sperm DNA damage	Governini et al. (134)
	PFOS, PFOA, PFNA, PFHxS PFHxA	Adulthood	No change in DNA integrity	Specht et al. (135) Emerce and Cetin (136)
	PFOS, PFOA, PFNA, PFHxS	Adulthood	No change in DNA methylation	Leter et al. (137)
**Semen volume**				
	PFOS, PFOA, PFNA, PFHxS	Adulthood	No change	Toft et al. (35) Joensen et al. (131) Joensen et al. (129) Vested et al. (130) Petersen et al. (132) Raymer et al. (133)
**Sperm motility**				
	PFOA	Adulthood	Increase	Toft et al. (35)
	PFOS, PFOA, PFHS	Adulthood	Decrease	Song et al. (78)
	Multiple PFOS, PFOA, PFNA, PFHxS	Adulthood	No change	Joensen et al. (131) Joensen et al. (129) Petersen et al. (132) Raymer et al. (133)
	PFOA, PFOS	*In utero*	No change	Vested et al. (130)
**Serum levels of testosterone**				
	PFHxS	*In utero*	Increase	Nian et al. (138)
	PFOS	Adulthood	Decrease	Joensen et al. (131)
	PFOA, PFOS, PFNA	Adulthood	Decrease	Cui et al. (139)
	PFOS, PFOA, PFHxS, PFNA	Adulthood	No change	Joensen et al. (129) Petersen et al. (132) Raymer et al. (133)
	PFOS, PFOA	*In utero*	No change	Vested et al. (130)
**Serum levels of sex hormone binding globulin**				
	PFOA	Adulthood	Increase	Petersen et al. (132)
	PFOA, PFOS, PFNA	Adulthood	Decrease	Cui et al. (139)
	PFOS, PFOA, PFHxS, PFNA	Adulthood	No change	Joensen et al. (129) Joensen et al. (131) Petersen et al. (132)
	PFOS, PFOA	*In utero*	No change	Vested et al. (130)
**Serum levels of luteinizing hormone**				
	PFOA, PFOS	Adulthood	Increase	Petersen et al. (132) Raymer et al. (133)
	PFOA	*In utero*	Increase	Vested et al. (130)
	PFBS, PFHpA	*In utero*	Decrease	Nian et al. (138)
	PFOS, PFOA, PFHxS	Adulthood	No change	Joensen et al. (129) Cui et al. (139)
	PFOS	*In utero*	No change	Vested et al. (130)
**Serum levels of follicle-stimulating hormone**				
	PFOA	*In utero*	Increase	Vested et al. (130)
	PFBS	*In utero*	Decrease	Nian et al. (138)
	PFOS, PFOA, PFHxS, PFNA	Adulthood	No change	Joensen et al. (129) Petersen et al. (132) Raymer et al. (133)
	PFOS	*In utero*	No change	Vested et al. (130)

The impact of PFAS on a variety of additional reproductive characteristics has also been investigated, including dysregulation of reproductive hormone profiles (141, 143, 144). In one such study, significantly lower serum testosterone levels were detected in male mice following 21 days of high (10 mg/kg) PFOS administration *via* oral gavage, compared to untreated controls (145). This finding supports previous evidence from studies of adult male rats exposed to PFOA by gavage at a concentration of 25 mg/kg/day for 14 days (146) and of mice treated for 28 days with PFOA (147). In the latter study, a dose-responsive reduction in testosterone and progesterone levels in the testis was revealed. These animal data are commensurate with some human investigations, which have also attributed reduced testosterone levels in men to high PFOS (131) and PFOA (139) levels. Furthermore, Luteinizing hormone (LH) and sex hormone binding globulin (SHBG) levels have been reported to correlate with increasing plasma PFOA concentrations in adult males (132, 133). Additionally, increased LH and follicle-stimulating hormone (FSH) were detected in men who experienced prenatal exposure to PFOA (130), indicating that this developmental phase may be particularly sensitive to maternal PFAS exposure. However, several conflicting studies fail to show any associations between PFAS exposure and plasma reproductive hormone levels in males (testosterone, LH, FSH, SHBG and estradiol) (129, 148, 149). Further, Nian et al. revealed a negative trend with FSH and PFBS in cord blood from newborns exposed to PFAS during pregnancy (138). As an additional caveat, animal study evidence should be interpreted cautiously as information on PFAS effects and male fertility is often not at environmentally relevant concentrations due to the much shorter half-life and faster elimination rates seen in animals, which result in lower internal levels at doses equivalent to human exposures.

### Mechanisms of PFAS Action on Reproductive Health

Testicular dysgenesis syndrome (TDS) is a term that encompasses a range of male reproductive disorders originating from fetal development (150) and which are thought to be a consequence of environmental influences (150–152). Such issues share similarities with those attributed to PFAS exposure, such as undescended testes (cryptorchidism), fertility issues in adults, and testicular cancer (150, 151). Fetal development of the male reproductive system is sensitive to disturbance by environmental factors such as diethylstilbestrol, a synthetic estrogen prescribed to pregnant women in the mid-1900s (153) and cyclooxygenase inhibitors such as ibuprofen and paracetamol (154). An increased incidence of cryptorchidism has been identified in men exposed to these environmental toxicants *in utero*, with responses being particularly pronounced in those exposed during the critical programming windows of the first and second trimester (155, 156). Studies in rats have proposed the existence of male programming windows corresponding to weeks 8-14 of pregnancy in humans during which androgen-induced masculinization occurs, including programming of testes descent. Thus, environmental exposures encountered during this period have the potential to affect normal male reproductive development and reproductive hormone balance (156), suggesting a mechanism by which PFAS exposure during gestation may impact subsequent developmental events within the male reproductive system.

**Figure 3:**
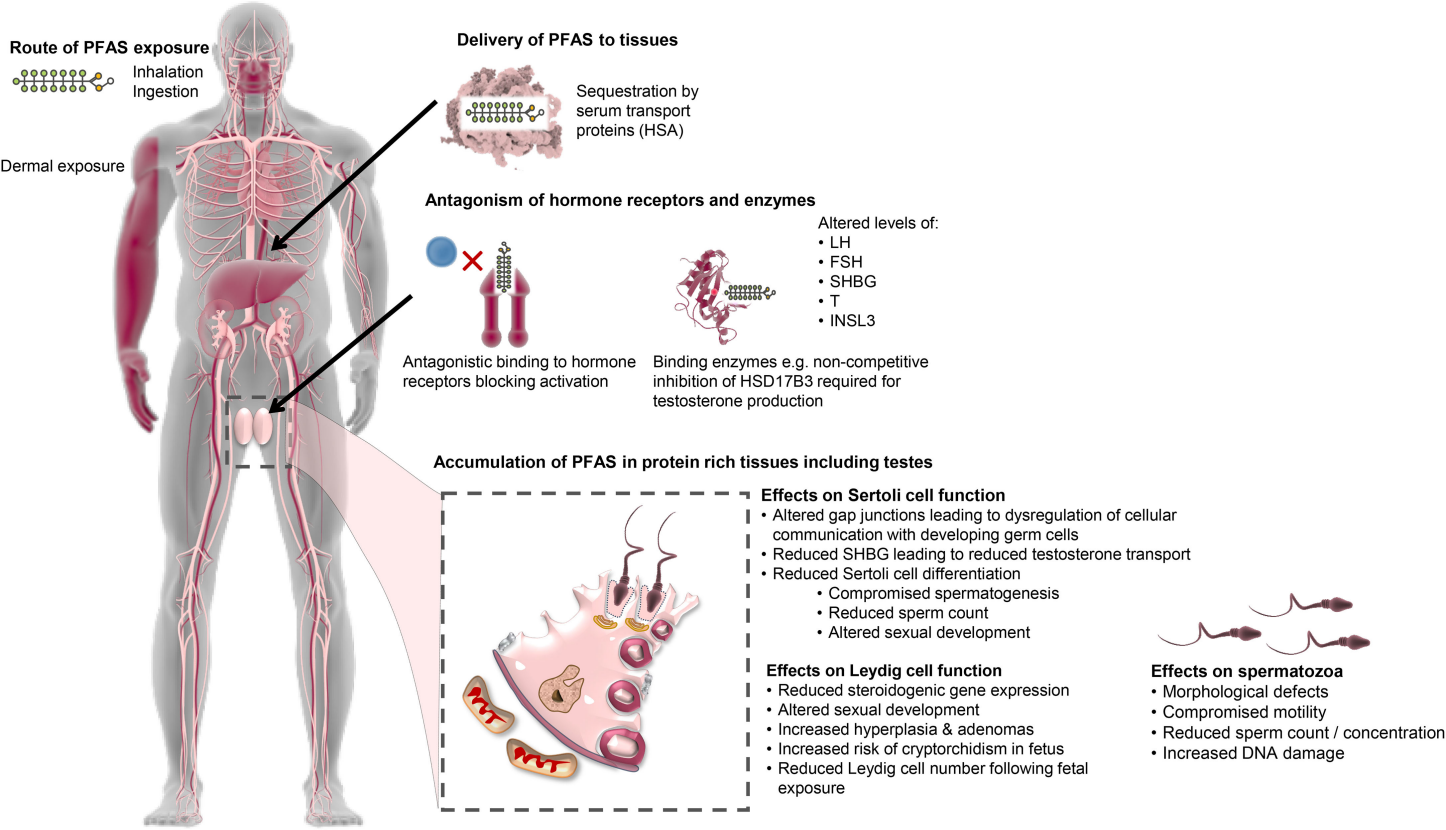
Proposed mechanisms of PFAS action pertaining to the male reproductive system. PFAS have the potential to enter the body through multiple routes. Following entry, PFAS are capable of binding to fatty acid binding proteins and transport proteins in the blood such as human serum albumin (HSA) and thereafter are thought to be transported throughout the body eliciting harmful endocrine effects *via* two possible mechanisms: disturbing steroidogenesis (e.g. *via* allosteric inhibition of vital enzymes) or directly interfering with steroid hormone receptors. This results in altered levels of reproductive hormones such as luteinizing hormone (LH), follicle stimulating hormone (FSH), sex hormone binding globulin (SHBG), testosterone (T) and insulin-like peptide 3 (INSL3), which has subsequent effects on male reproductive processes. PFAS also accumulate in protein rich tissues, including the testes, which is facilitated by the high expression of fatty acid binding proteins. Here, PFAS impact testicular cell function, namely Leydig and Sertoli cells. Altered Leydig cell function leads to reduced testosterone production resulting in altered sexual development, increased incidence of hyperplasia and adenomas and increased risk of cryptorchidism in the fetus. This reduction in testosterone leads to attendant impacts on Sertoli cell function by reducing Sertoli cell differentiation and precipitating compromise of spermatogenesis, reduced sperm count and altered sexual development. Gap junctions between Sertoli cells and developing germ cells are also affected by PFAS, which reduces communication between the cells, negatively affecting spermatogenesis and resulting in a range of defects in the mature spermatozoa.

Endocrine disruptors are chemicals (both naturally occurring and synthetic) that interrupt the normal hormonal system of the body, either through direct hindrance of hormonal pathways or through mimicking the hormones within the endocrine system (157, 158). This can result in variable consequences such as dysregulation of immune, reproductive and developmental pathways (159–162). It follows that this diverse group of chemicals have been widely implicated in the development of reproductive abnormalities including TDS (150, 163–166). Indeed, the TDS hypothesis suggests that *in utero* exposure to endocrine disruptors damages testis development resulting in decreased function in adulthood, with symptoms ranging from moderately reduced semen quality through to the promulgation of testicular cancer (6, 167). PFAS display properties consistent with that expected of endocrine disruptors (1, 6, 168), and are widely reported as having endocrine disrupting actions (1, 6, 168–171). Accordingly, PFAS exposure often results in altered androgen and insulin-like factor 3 (INSL3) dependent processes (172–175) (**Figure 3**). There are two suggested mechanisms by which PFAS produce harmful endocrine effects: either by disturbing steroidogenesis (6) or by interfering with steroid hormone receptors (176, 177).

Specifically, at least some of the pathologies attributed to *in utero* PFAS exposure are hypothesized to arise due to abnormal Leydig cell development and/or function (6, 176). Leydig cells are a vital component of the male reproductive system responsible for synthesizing the steroid hormone testosterone, which is essential for sexual development and testis decedent in the fetal period (178), and the support of normal sperm production in the adult (179). Biegel et al. reported altered Leydig cell function *in vitro* with cells isolated from untreated rats, in which a dose-dependent decrease in testosterone was seen following a 5-hour treatment with PFOA (IC50 approximately 200 μM) (146). Additionally, *ex vivo* investigations with Leydig cells isolated from rats gavaged for 14 days with 25 mg/kg/day of PFOA showed these alterations are reversible following cessation of PFOA treatment (146). Such effects may be attributed to PFAS interfering with one, or more, of the enzymes involved in steroidogenesis. By way of example, PFAS may directly inhibit the catalytic activity of the 3β-hydroxysteroid dehydrogenase (HSD3B) enzyme by competing against its native pregnenolone substrate, thereby limiting the production of testosterone in rat Leydig cells (180). In humans, PFOS displays non-competitive inhibition of HSD17B3, another enzyme required for testosterone synthesis (176). Interference with steroid hormone receptors has also been documented, with the binding of PFAS leading to antagonism of androgen receptors (50), thereby blocking their activation by androgens, such as testosterone, in a dose-dependent manner (181). Other studies have reported a reduction in fetal Leydig cell number in male offspring exposed *in utero*, with mothers gavaged with 5 or 20 mg/kg of PFOS daily from gestational day 11 to 19 (182), which provides a tenable explanation for the reduction in testosterone levels seen in independent studies of rat models (146). Alternatively, elevated exposure to PFAS has been positively correlated with increased serum cholesterol in humans (1), which may lead to an increase in the production of steroid hormones. Of concern, the consequences of alterations resulting from prenatal PFAS exposure have the potential to be passed on to offspring through epigenetic transgenerational inheritance modalities (183) and may thus increase the susceptibility of future generations to disease, as demonstrated with other environmental factors (121, 184).

Further to this, at least two studies have shown that high PFOS exposure in adult men results in a higher proportion of morphologically abnormal sperm cells (35, 129). However, Vested et al. failed to identify any such association between PFOA exposure *in utero* and the proportion of morphologically normal spermatozoa (130). The authors did, however, report associations between PFOA exposure and total sperm count and concentration (130), which would suggest that the timing of exposure plays a part in the mechanism by which PFAS affects the fidelity of sperm production, that is; whether PFAS exposure gives rise to defects in sperm morphology or sperm count. This reasoning is plausible since sperm morphology and motility are primarily determined during sperm production and maturation in adulthood. In contrast, the capacity for sperm production is determined during the fetal period of sexual organ development (130). Furthermore, the relationship between sperm count/concentration and PFOA exposure suggests an effect on Sertoli cell development during the fetal period, as failure of Sertoli cell maturation and consequential inability to support spermatogenesis invariably results in lower rates of sperm cell production (185). This is supported by evidence demonstrating that *in vitro* PFAS exposure disturbs Sertoli cell function by altering the gap junction network with implications for the intracellular communication and cell-cell interactions (186–188) that are necessary for the support of spermatogenesis (189–192). Abnormal Sertoli cell development during the initial *in utero* stages of male reproductive tract development will also, in turn, impact Leydig cell function and subsequent masculinization events (193).

An *in vitro* study using a human stem cell model of spermatogenesis discovered a reduction in both spermatogonia and primary spermatocyte markers when cultures were treated with a mixture of PFOA, PFOS, and PFNA at levels consistent with general population exposure and occupationally exposed individuals, suggesting a potential long-term effect on fertility through exhausting the spermatogonial stem cell pool, rather than directly affecting cell viability (194). In agreement with this notion, an *in vivo* study in mice revealed a reduction in sperm count and testicular weight upon treatment with PFOS over five weeks (195), while in a zebrafish study the gonadal structure of juvenile males was altered by a 5-month treatment period with PFOS, ultimately resulting in fewer spermatogonia (196). Such results have been attributed to a combination of increased apoptosis and reduced proliferation of germ cells (195). However, the effects of PFAS exposure on the reproductive system differ depending on the specific PFAS and the toxicokinetics of the species studied (197). For example, PFOA mainly accumulates in the plasma and liver of rats, with the half-life in female rats at 1 day being much shorter than in males at 15 days (106). By comparison, high PFOS accumulation has been detected in the liver and lungs of humans (20, 198), with no differences in elimination between genders (12). Another mechanism by which PFAS mediated testicular issues may arise is through the binding of fatty acid binding-proteins (FABP), a family of proteins that bind fatty acids to enhance their solubility, and to aid in both the intracellular and extracellular transport of fatty acids (199). The most common FABP is albumin, which binds and transports fatty acids within the plasma and interstitial fluid (200, 201). There are distinct types of FABP, with each type exhibiting certain tissue distribution patterns and named accordingly (202). For example, mammalian testicular cells express high levels of the *Fabp9* gene (also known as testes FABP) (202), and *Fabp12* gene expression has been detected in adult rat testis (203). Due to the distinct distribution and expression patterns of FABP encoding loci, it is suggested they may play a role in cell proliferation and differentiation (199, 204), specifically spermatogenesis in the testes (203). Therefore, the testes are a vulnerable organ for PFAS-mediated damage, owing to their abundant expression of FABPs that have a propensity to bind the perfluoroalkyl chains and lead to sequestration of PFAS.

## Remediation of Environmental PFAS Contamination

**Figure 4:**
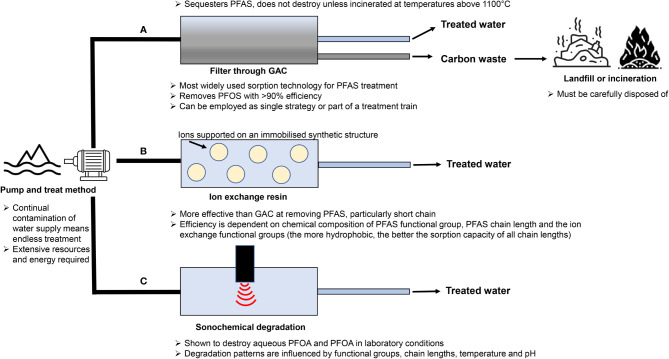
Schematic diagram of three possible treatment mechanisms for PFAS contaminated water. **(A)** Carbon-rich sorbents such as granular activated carbon (GAC) have a long history of being utilised to remove a variety of organic contaminants from water and as such are by far the best studied and most widely used sorption technology for treating PFAS contaminated water sources (208). Granular activated carbon has been shown to reliably remove PFOS with over 90% efficiency (59, 210) and is thus now the reference point for comparison of all new PFAS water sorption technologies. This technology can be employed to treat water before it reaches consumers, either as a single strategy, or as part of an integrated treatment programme. This treatment often involves the pump and treat method in which groundwater is extracted and filtered (208, 211), with the sorbent then being disposed of in landfill sites, provided certain risk criteria are met and the chemicals remain sequestered. International conventions state that waste materials containing > 50 mg/kg of PFAS must be treated in such a way as to destroy these chemicals, which is often accomplished by incinerating at high temperatures (over 1100°C) (208, 212). **(B)** Ion exchange uses anion exchange to target a wider range of PFAS, allowing for more efficient removal. Removal occurs *via* electrostatic interactions between the charged functional groups of PFAS chemicals and ions supported on an immobilized synthetic structure (213). In comparison to activated carbon treatment, this method has been shown to be more effective in removing PFAS, particularly the short chain variants (205). However, the efficiency of ion exchange technologies is dependent on several factors, such as the chemical composition of PFAS functional group, PFAS chain length and the ion exchange functional groups (the more hydrophobic, the better the sorption capacity of all chain lengths) (213). The success of almost all remediation strategies employed has been shown to depend on the perfluoroalkyl chain length, with increased efficacy seen with smaller chain length (214). **(C)** However, simply removing PFAS from water does not destroy the chemical, hence why further processing or incineration is required, making removal techniques lengthy and potentially hazardous. Sonochemical degradation has been shown to destroy aqueous PFOA and PFOA in laboratory conditions (213), demonstrating sonic irradiation can be effectively employed to reduce PFAS contamination at environmentally relevant levels (215). The degradation patterns of PFAS chemicals are influenced by their functional groups and chain lengths as well as physical variables such as temperature and pH (216). Utilizing the method of groundwater pumping is seen as a potentially endless endeavor due to continual contamination from untreated water sources. This, in turn, raises questions as to whether the pump and treat method is sustainable in the long-term treatment of PFAS contamination due to extensive resources and energy required (208).

Extensive worldwide use of PFAS has led to pervasive contamination of land and water, which demand remediation if we are to have any prospect of combating the adverse health outcomes attributed to these chemicals, both in humans and wildlife. Environmental matrices that require targeting for remediation include groundwater, drinking and surface water, as well as soil and sediments (66, 205). However, this diversity of substrates provides unique challenges considering that different PFAS do not all interact with different matrices in the same manner (206), nor do they behave like other environmental contaminants (207). It is thus imperative that chemical features specific to PFAS are taken into consideration when designing remediation strategies to ensure thorough and long-lasting removal is achieved. Similarly, the logistics of the treatment, accessibility, and safety measures need to be taken into consideration. Regrettably, options for PFAS remediation remain limited, with most current technologies having originally been developed for the removal of other contaminants (208). Thus, there is considerable scope for the development of novel technologies to facilitate PFAS remediation, perhaps even employing a combination of treatment processes tailored to the site and/or PFAS profile to achieve the most cost-effective and efficient treatment process for each site (209). Illustrated in **Figure 4** are three remediation strategies that can be employed for effective treatment of PFAS water contamination (210–216). A novel technique has been proposed that utilizes plant proteins for effective removal of PFAS water contamination, through the pump-and-treat method (217). Such proteins contain both charged and uncharged residues on their surface, which allows them to form bonds with ligands through electrostatic interactions and/or hydrophobic/hydrophilic interactions (218), providing a method by which they could potentially remove PFAS contamination from water. Previous studies have shown PFOS forms a strong salt bridge with HSA, resulting in a high adsorption ratio of 45:1 PFOS to HSA (219). Building on these observations, Turner et al. (217) investigated the sequestration efficacy of six plant protein isolates and found that hemp protein has the highest removal rate for total PFAS at 92.5%, along with soy and pea proteins (around 82%), which is comparable to the granular activated carbon technique (95%). This technique provides a means by which plant proteins could be employed to reduce PFAS contamination within the body and thus warrants further investigation.

## Removing PFAS Accumulation in the Human Body

Unlike other environmental toxicants, such as parabens (220), the body cannot metabolize or facilitate the rapid removal of PFAS. Thus far, there has been limited investigation into the possibility of sequestering PFAS chemicals to reduce bioaccumulation within contaminated individuals in order to mitigate negative health outcomes. However, the adaption of existing techniques developed for other environmental contaminants may provide opportunities by which to reduce PFAS accumulation. Unfortunately, most of these techniques are only newly recognized and as such are still at an early stage of research with inadequate evidence of efficacy (221).

One process investigated for detoxification in humans is the exploitation of the body’s natural excretion through perspiration, with an assortment of toxicants shown to be excreted in this manner, such as metals (222), phthalates (223) and bisphenol A (224). Studies have reported that induced perspiration treatment in individuals with toxicant accumulation, specifically polychlorinated biphenyls (PCBs) compounds, results in a statistically significant reduction in body burden (225, 226). Although promising, based on the limited available evidence, it seems that PFAS may not be readily excreted through sweat (227). Conversely, shorter half-lives are observed in women, which are proposed to result from increased PFAS elimination through menstruation, pregnancy or lactation (1, 109, 228). Accordingly, studies have shown regular phlebotomy or blood donation can also increase the elimination of PFAS (229, 230). Lorber et al. showed males undergoing regular blood withdrawals had 40% lower PFHxS, PFOA and PFOS levels in comparison to males in the general population (231), and suggested a 9% reduction in circulating blood volume was required to achieve significant reductions in PFAS levels. Additionally, evidence shows that bile acid sequestrants, such as cholestyramine, bind to PFAS toxicants within the gastrointestinal tract, thus preventing enterohepatic recirculation and increasing excretion of cholesterol and PFAS (227, 232). One case study with a single individual with high PFAS contamination showed increased levels of PFAS in stool samples following treatment with cholestyramine, with serum levels subsequently reducing with continued treatment (227). Ducatman and colleagues recently substantiated this evidence by analyzing the C8 Health Project Data (43) in which they found a reduction in serum PFAS levels (especially PFOS from 19 to 1 ng/mL) in individuals reporting to take regular cholestyramine medication, although data on duration and medication dosage administered was not reported (233). Of course, additional investigations are required to further corroborate these findings, but thus far evidence from these studies implies that utilizing cholestyramine treatment may allow for enhanced toxicant elimination.

## Conclusion

Increasing awareness of the potential health implications of PFAS and realization of the extent of environmental contamination has led to a rising demand for research into definitive health risks and effective remediation strategies. Animal models have been widely employed to investigate *in vitro* and *in vivo* consequences of PFAS exposure, as well as the toxicology of these chemicals. Such studies complement a growing body of evidence from human epidemiological studies. However, the literature abounds with conflicting evidence, and as such, it remains challenging to draw accurate conclusions regarding the causality of PFAS related health issues. This situation is exacerbated by the repeated demonstration that outcomes differ depending on factors such as the specific PFAS chemical(s) (of which there are over 4,700), stage of development (i.e., during fetal development or in later life) and duration of exposure, level and mix of contamination, route of exposure, and interaction with other environmental contaminants and toxicants, all of which are influenced by geographical location. These factors present significant difficulties for researchers in planning, executing, and interpreting studies, and thus hinder our ability to directly compare PFAS exposure studies. While standardization therefore remains an essential priority for future research, the identification of appropriate cellular model(s) with which to directly investigate and unlock the interaction of PFAS with the male reproductive system would also be advantageous. In addition, agreement is needed regarding endpoint measures, in which subtle changes, such as decreases in fertility or metabolic sequelae, may be used as early markers of PFAS-mediated health effects, rather than more extreme factors such as tumors. In this regard, the male reproductive system offers notable advantages as a sensitive marker of human disease and may ultimately provide a unique opportunity for assessing the emerging threat to human health posed by PFAS exposure. Indeed, this model draws on a growing body of evidence of a strong association between a male’s general health and reproductive potential, with infertility being strongly correlated with future health concerns such as testicular cancer, ischemic heart disease and diabetes.

## Future Perspectives

We contend that the identification of a reliable indicator of PFAS exposure would allow for the identification of reproductive health conditions resulting from PFAS bioaccumulation and aid in identifying, with certainty, the mechanisms by which PFAS impacts male reproductive health. Exploiting male reproductive function and sperm biology as a non-invasive means by which to investigate health outcomes is justified due to the responsiveness and sensitivity of the male reproductive system to environmental toxicants. Indeed, previous studies have employed this system as a marker to define the health effects of environmental factors such as acrylamide (234, 235), mobile phone radiation (236), and heat (237, 238). Additionally, the male reproductive system is known to be an early indicator for the onset of chronic diseases such as coronary and inflammatory diseases, making it a suitable indicator of general health. Strong associations have been seen between PFAS exposure and testicular dysfunction, indicating the male reproductive system is vulnerable to PFAS-mediated damage. In this context, perhaps the most suitable human cohorts to study are those that have received occupational PFAS exposure, such as firefighters, to determine the common effects of exposure and gain insight into possible mechanisms of action. It would then be pertinent to study individuals with idiopathic infertility to identify if any clear associations can be drawn between their diagnosis and PFAS levels, through assessment of detailed life history and daily routine/exposure information. These factors could then be used to screen for affected individuals within the general population. In addition to elucidating the toxicological effects of PFAS chemicals in humans, there is a need for data on a wider range of PFAS in order to regulate, legislate and ban those that are harmful, thus preventing further contamination from replacement PFAS, which may be just as harmful as the legacy variants.

## Author Contributions

LC conceived of the idea and wrote the first draft of the manuscript. MG, GI, MD, BT, BC, AE, SR and BN conceived of the idea, sourced funding, and edited the manuscript. All authors approved the final version of the manuscript.

## Funding

This work was supported by funding from a National Health & Medical Research Council of Australia (NHMRC) Targeted Call for Research into Per- and Poly-Fluoroalkylated Substances (APP1189415) awarded to BN, MG, GI, MD, BT, BC, AE, and SR. BN is the recipitent of an NHMRC Senior Research Fellowship (APP1154837). MD is the recipient of an NHMRC Investigator Grant (APP1173892) and a Defeat DIPG ChadTough New Investigator Fellowship.

## Conflict of Interest

The authors declare that the research was conducted in the absence of any commercial or financial relationships that could be construed as a potential conflict of interest.

## Publisher’s Note

All claims expressed in this article are solely those of the authors and do not necessarily represent those of their affiliated organizations, or those of the publisher, the editors and the reviewers. Any product that may be evaluated in this article, or claim that may be made by its manufacturer, is not guaranteed or endorsed by the publisher.
